# A retrospective clinical study of 98 adult idiopathic primary intraventricular hemorrhage cases

**DOI:** 10.1097/MD.0000000000005089

**Published:** 2016-10-21

**Authors:** Rui Guo, Lu Ma, Bal Krishna Shrestha, Zhiyuan Yu, Hao Li, Chao You

**Affiliations:** Department of Neurosurgery, West China Hospital, Sichuan University, Chengdu, Sichuan, P.R. China.

**Keywords:** etiology, hypertension, intracerebral hemorrhage, primary intraventricular hemorrhage, prognosis, stroke, treatment

## Abstract

The aim of the study is to define the clinical features, risk factors, treatment and prognosis of idiopathic primary intraventricular hemorrhage (IPIVH).

We retrospectively collected the data of consecutively admitted patients who were diagnosed and treated for IPIVH in our hospital from January 2010 to December 2014. The clinical information, treatment, and prognosis at the 6-month follow-up were analyzed.

Among the 3798 cases of spontaneous intracranial hemorrhage (ICH), 98 IPIVH (2.58%) patients were recruited for the study. The study population consisted of 60 males and 38 females, with an average age (± standard deviation, SD) of 51.20 ± 15.48 years. The initial symptoms were headache (75 cases) and impaired consciousness (23 cases). The surgical treatments included hematoma evacuation under a microscope or an endoscope in 8 cases (8.16%), external ventricular drainage (EVD) in 11 cases (11.22%), lumbar drainage (LD) in 10 cases (10.20%), and a combination of EVD and LD in 11 cases (11.22%). In total, 4 patients died in the hospital (4.08%). At the 6-month follow-up, 73 patients (74.49%) had an improved outcome (modified Rankin scale [mRS] < 3), and 21 patients (21.43%) had a poor outcome (mRS ≥ 3 points) at the end of the 6-month follow-up.

IPIVH is rare in clinical practice, and hypertension is the most common risk factor. Furthermore, the treatment of IPIVH is still controversial. Hematoma evacuation under a microscope or an endoscope, EVD, LD and a combination of EVD and LD could be surgical options for the treatment of IPIVH patients. The outcomes for IPIVH patients could be relatively favorable with individualized treatment.

## 1 Introduction

Intraventricular hemorrhage (IVH) is a common complication of intracerebral hemorrhage (ICH) or subarachnoid hemorrhage (SAH). However, it has been reported that 3.1% to 9% of ICH cases are primary intraventricular hemorrhage (PIVH) without parenchymal involvement.^[1,2]^ We define idiopathic primary intraventricular hemorrhage (IPIVH) as PIVH without structural cerebrovascular abnormalities, such as thalamus hemorrhage, arteriovenous malformations (AVM), aneurysms, moyamoya disease, and apoplexy.

**Figure 1:**
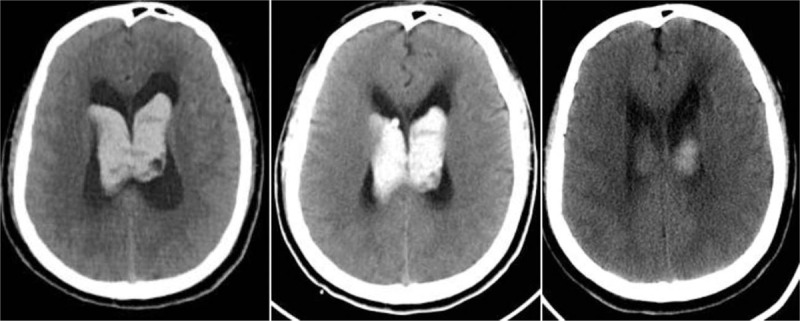
CT scan of an IPIVH patient at admission, 1 day after EVD and at discharge. CT = computed tomography, EVD = external ventricular drainage, IPIVH = idiopathic primary intraventricular hemorrhage.

Patients with IPIVH often present with sudden onset of headache, nausea, or vomiting with/without impaired consciousness. Focal neurological signs are usually minimal or absent among these patients.^[3]^ Therefore, before the emergence of modern imaging techniques, IPIVH was considered a fatal disease because it could be diagnosed only through autopsy. Modern brain imaging techniques have simplified the diagnosis of IPIVH (Fig. 1); however, relatively little is known about its clinical features, risk factors, treatment, and prognosis. Most IVH studies have included secondary or traumatic bleeding,^[4,5]^ which cannot explain the clinical features of IPIVH. Moreover, the sample sizes of these studies were generally small.^[1,3]^ In our study, we utilized a retrospective cohort at West China Hospital from January 2010 to December 2014 to define the clinical features, risk factors, treatment, and prognosis of PIVH.

## 2 Materials and methods

We retrospectively searched the records of all patients with a discharge diagnosis of IVH who were admitted to the Department of Neurosurgery at West China Hospital from January 2010 to December 2014. Approval for the retrospective medical record review and imaging review was obtained from the Institutional Review Board at the West China Hospital of Sichuan University.

We diagnosed patients with PIVH when computed tomography (CT) revealed hemorrhage restricted to the ventricles. Patients with extravasation of blood from disrupted or ruptured brain parenchyma or discernible SAH on CT scans were excluded. After the identification of 148 patients with PIVH, 50 cases were excluded due to underlying causes such as moyamoya disease, AVM, aneurysms, brain tumors, and trauma confirmed using digital subtraction angiography (DSA), computed tomography angiography (CTA) or magnetic resonance angiography (MRA) or because medical information was unavailable. Finally, 98 IPIVH patients were retrospectively included in this analysis.

We extracted the clinical data from the patient's medical records including gender, age, risk factors, concomitant diseases (hypertension, diabetes, hyperlipidemia, smoking history, drinking history, family history of stroke, and coagulation disorders), clinical manifestations, abnormal neurological findings, treatment, and medical complications. The level of consciousness at admission was evaluated using the Glasgow Coma Scale (GCS). All CT scans of IPIVH patients were reviewed by the members of our study group, and IVH was evaluated using the Graeb score.^[6]^ The modified Rankin scale (mRS) was applied to measure neurological disability at admission and at the end of the 6^-^month follow-up. Patients with a mRS score < 3 were defined as independent, and a mRS score ≥ 3 indicated a disability.^[7]^ The demographic characteristics, risk factors, clinical events, and outcomes were analyzed using a chi-square test, Student's *t* test or Mann–Whitney *U* test. Predictors of in-hospital mortality and poor outcomes were investigated using logistic regression. Analyses were performed using SPSS version 21 (IBM Corp., Armonk, NY). A value of *P* < 0.05 was considered statistically significant.

## 3 Results

### 3.1 Patient characteristics

From January 2010 to December 2014, 3798 cases of spontaneous ICH were diagnosed in our hospital, 353 of which were secondary IVH. IPIVH accounted for only 2.58% of all the ICH cases in our study. As shown in Table 1, the average age (± standard deviation, SD) of the IPIVH patients was 51.20 ± 15.48 years. The study included 60 males and 38 females, with a male-to-female ratio of 1.58:1. In addition, no significant difference was observed for the age of onset between males (50.97 ± 15.31 years) and females (51.58 ± 15.95 years) (*P* = 0.85). All patients underwent at least 1 vascular imaging examination (DSA 64.29%, CTA 32.65%, or MRA 8.16%). The most common risk factor in our study was hypertension (62.24%). The average GCS (± SD) was 11.72 ± 3.06 at admission. The average Graeb score according to the emergency CT scan was 5.51 ± 2.76. Some degree of hydrocephalus was observed in the CT scan of 45 patients at admission.

**Table 1:**
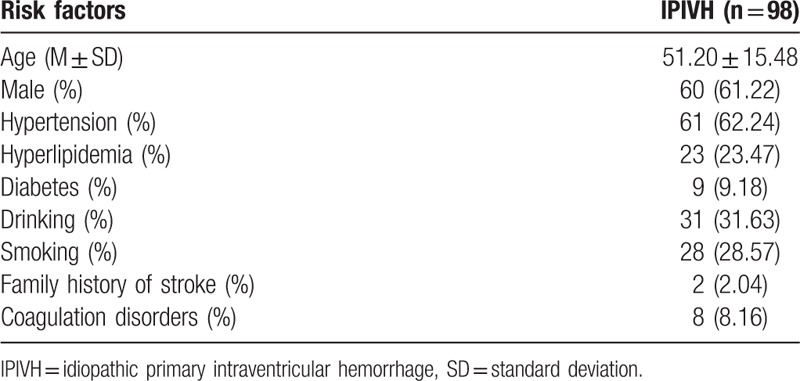
Comparison of demographic and clinical characteristics.

Headache with/without nausea or vomiting was experienced by 75 patients, and nuchal rigidity was present in 31 patients. All the 32 noncomatose patients with hydrocephalus presented with headache. Among the patients with a Graeb score > 6, 92.31% (36/39) presented with a headache. Impaired consciousness was observed in 23 patients. Focal motor symptoms were absent (75 patients) or mild (23 patients), and unilateral or bilateral extensor plantar reflexes were positive in 12 patients. Neuro-ophthalmological abnormalities were recorded in 15 patients.

### 3.2 Surgery versus observation

The recommended treatment was discussed by a board that consisted of cerebrovascular surgeons and stroke neurologists. Any treatment was undertaken after informed consent was obtained from the patients or appropriate surrogates. Generally, at our institution, surgery is performed after the comprehensive consideration of the following clinical presentations and imaging results: impaired consciousness (particularly a GCS score ≤ 8), worsening of clinical status, a relatively large amount of blood in the ventricular system, and acute hydrocephalus.

In the present study, the surgical treatments included hematoma evacuation (craniotomy with/without external ventricular drainage [EVD]) in 8 cases (8.16%), EVD in 11 cases (11.22%), lumbar drainage (LD) in 10 cases (10.20%), and a combination of EVD and LD in 11 cases (11.22%).

### 3.3 Outcomes and complications

Overall, 4 patients died during hospitalization (4.08%). The median duration of the hospital stay was 14.5 days. The mean mRS score for the entire cohort was 2.67 ± 1.07 at presentation, which decreased to 1.40 ± 1.38 at the time of the last follow-up evaluation (Table 2). Of the 40 patients who received surgery, 31 patients presented with neurological improvement or no functional improvement, whereas 9 patients experienced functional deterioration or died. Among the 58 patients who did not receive surgery, 41 patients presented with neurological improvement or no functional improvement by the end of the 6-month follow-up. In the logistic regression analysis, no independent predictors of inpatient deaths were found (Table 3). However, the admission GCS score was the only independent predictor of the outcome at the 6-month follow-up in the discharged IPIVH patients (Table 4).

**Table 2:**
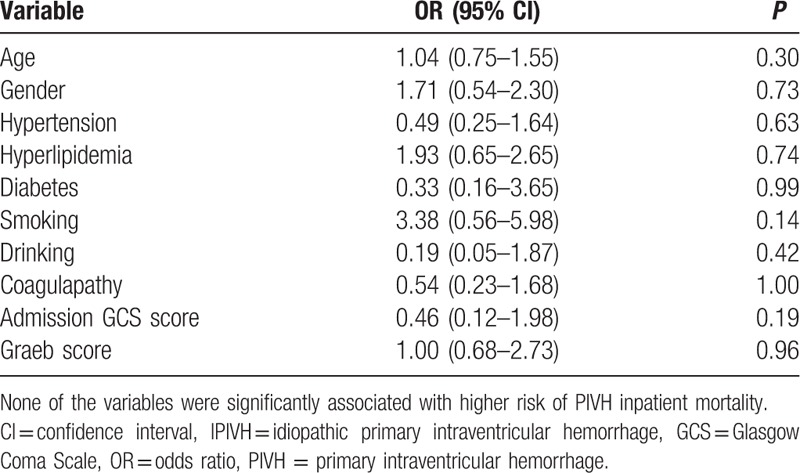
Logistic regression analysis results of idiopathic primary intraventricular hemorrhage (IPIVH) inpatient mortality predictors.

**Table 3:**
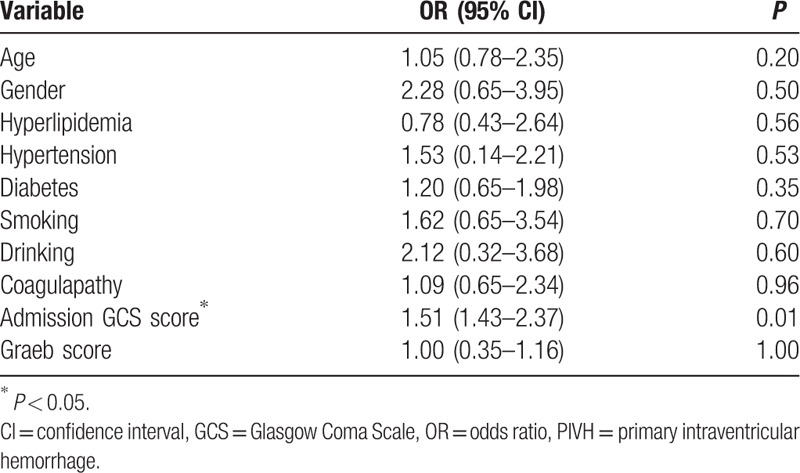
Logistic regression analysis results of discharged PIVH patients’ outcome predictors at 6th month.

**Table 4:**
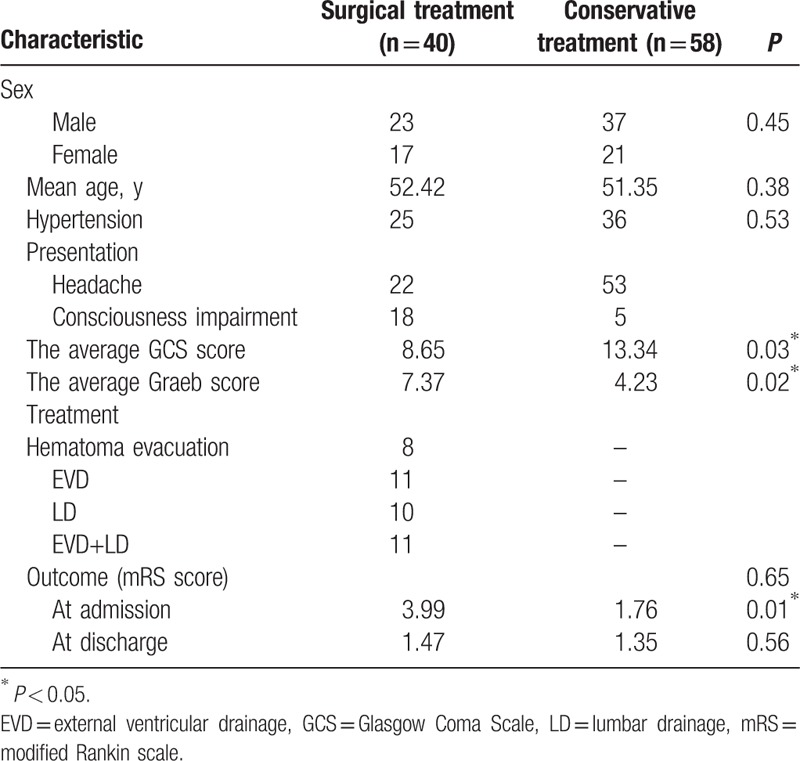
Comparison of surgical treatment and conservative treatment.

Pulmonary infection was the most common complication in our study, which occurred in 22 patients (22.45%). Hydrocephalus was observed in the follow-up CT scans of 14 patients (14.29%). In addition, 2 patients (2.04%) experienced intracranial infection in the surgery group. The details of the complications in each group are shown in Table 5.

**Table 5:**
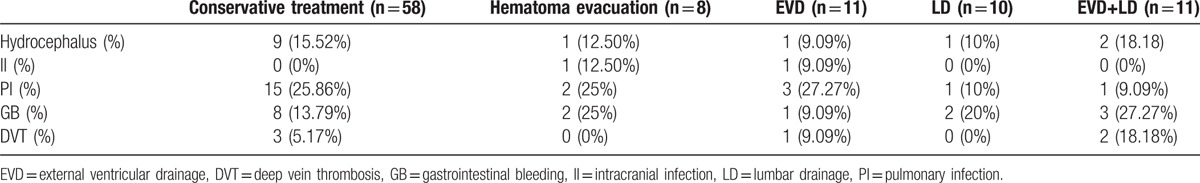
Complications in each group.

## 4 Discussion

Spontaneous ICH is an important global public health problem that leads to high rates of mortality and disability.^[8]^ Society and the environment^[9,10]^ have a great impact on the incidence and prognosis of diseases including ICH. Studies have indicated that socioeconomic factors such as income inequalities can lead to different incidences of ICH.^[11,12]^ In addition, seasonal changes and geographical variations can also influence the prevalence of stroke.^[13,14]^ Additionally, people make different medical decisions based on the health care system and cultural differences,^[15,16]^ which may lead to variations in prognosis. Although ICH accounts for 2 million patients worldwide each year.^[8]^ IPIVH is an uncommon type of ICH and constituted only 2.58% of all ICH cases in our series. The present study defined the clinical features, risk factors, treatment, and prognosis of IPIVH.

Our series included 60 males and 38 females, with a male to female ratio of 1.58:1, whereas the ratio varied between from 0.56 to 1.40 in other studies.^[17–20]^ Hypertension was the most common risk factor (61/98, 62%) in our study. However, the frequency of hypertension varied in previous studies from 29% to 100%.^[3,11–22]^ Martí-Fàbregas et al^[18]^ stated that hypertension can induce hemorrhage in choroidal arteries as it does in other arteries, which leads to IPIVH. Hyperlipidemia was recorded in 23 patients (23.47%). Although ICH has been reported to be associated with dyslipidemia in some studies,^[23,24]^ the relationship between serum cholesterol levels and increased risk of ICH is still controversial.^[25,26]^ Diabetes occurred in 9.18% of our study population compared to 0%,^[14]^ 8%,^[19]^ and 33%^[27]^ in other series. Hyperglycemia may increase the risk of hemorrhage through inflammatory^[28]^ and toxic^[29]^ effects of oxygen-free radical generation. Besides, Khealani reported the possible correlation between immunodeficiency and PIVH.^[30]^ However, none of our patients were found to be human immunodeficiency virus (HIV) positive.

Headache, which was likely secondary to chemical stimulation and the mass effects of the hematoma, was experienced by 75 patients (77%) compared to 78% in the series by Angelopoulos et al.^[17]^ Impaired consciousness was observed in 23 of our patients, which may be due to brainstem or bilateral hemispheric dysfunction. Impaired consciousness was also reported in previous studies.^[17,31]^ Nuchal rigidity and unilateral or bilateral extensor plantar reflexes were frequently recorded in the present study. However, focal motor symptoms were absent or mild. The absence of focal motor symptoms was likely due to midline hemorrhage without parenchymal involvement, whereas mild focal motor symptoms can be explained by asymmetrical bleeding in the ventricles.

Due to the absence of evidence-based guidelines,^[32]^ the management of IPIVH remains controversial. A previous study^[33]^ suggested that the treatment should be targeted to the early clearance of blood from the ventricular system. EVD, LD, and hematoma evacuation under a microscope or an endoscope were applied in our series. EVD is necessary in cases of acute hydrocephalus. However, the precise clinical and radiological indications for EVD have not been clarified.^[34]^ Furthermore, it has been suggested that EVD is sometimes ineffective due to catheter obstruction by blood and could even decrease the rate of clot resolution by removing the tissue plasminogen activator released from the clot. However, the concomitant use of intraventricular antifibrinolytics, such as tissue recombinant plasminogen activator (rtPA) or urokinase, has been shown to be safe and effective in response to the complications of EVD. Case series have demonstrated improved outcomes with urokinase.^[35,36]^ However, due to the safety concerns of urokinase, rtPA has become the thrombolytic choice. The Clot Lysis: Evaluating Accelerated Resolution of Intraventricular Hemorrhage (CLEAR-IVH) trial^[37]^ showed that rtPA can accelerate the resolution of IVH. Furthermore, a meta-analysis including 4 randomized and 8 observational studies also indicated that intraventricular fibrinolysis (IVF) was associated with a decrease in mortality and an increase in good functional outcomes.^[38]^ However, the Clot Lysis: Evaluating Accelerated Resolution of Intraventricular Hemorrhage Phase III (CLEAR III) trial demonstrated that most IVH patients could not achieve a substantial reduction of hemorrhage with rtPA (1.0 mg every 8 hours).^[39]^ Although the conventional treatment for IVH is EVD, several studies have investigated the feasibility of surgical evacuation such as endoscopy and minimally invasive surgeries (MIS) for the treatment of IVH. Surgical evacuation was reported to have favorable results in some observational studies^[40,41]^; however, randomized clinical trials are still needed to prove its efficacy over conventional treatments. Furthermore, LD, which has been shown to be a promising treatment strategy,^[42,43]^ should also be considered a treatment option for IPIVH.

In our study, the long-term outcomes were relatively favorable for IPIVH, with a mean mRS score of 1.40 ± 1.38 at the 6-month follow-up. The in-hospital mortality in our study was 4%, which was inconsistent with the results reported in previous studies (20–46%).^[1,3,17,44]^ A previous study has shown an association between IPIVH prognosis and the amount of hemorrhage.^[45]^ However, we and other researchers^[1,17,18]^ failed to find such a correlation. Our cohort indicated that the level of consciousness at admission was an important prognostic factor, which was consistent with other series.^[17,45]^ The patients who died in our study were all found to have impaired consciousness at admission. We also found that pulmonary infections affected 22.45% of IPIVH patients, which was consistent with the general incidence of stroke-associated pneumonia.^[46]^ Similar to other studies, we found that hydrocephalus was also one of the most common complications.^[18,19,45]^ Hydrocephalus may be caused by the obstruction of cerebrospinal fluid circulation or a decrease in the absorption capacity of the meninges. This condition can relieve itself in most cases, but sometimes a surgical shunt^[6]^ is needed.

Our study has several limitations. Although our series is one of the largest samples of IPVIH patients, the relatively small sample size at a single institution may limit the generalizability of our results. In addition, the retrospective nature of this study may inevitably introduce sampling bias. Another important limitation is that our logistic regression model included limited variables. Other factors that were not included in our model might play important roles in predicting the outcome of IPIVH. Finally, some of the included patients received >1 type of surgical treatment; therefore, it is difficult to make comparisons between the various surgical procedures.

In summary, our study showed that IPIVH was relatively rare in clinical practice. Furthermore, the GCS score at admission was an independent predictor of the outcome of the discharged patients at the 6-month follow-up. Treatment of IPIVH still requires further investigation. A craniotomy under a microscope or an endoscope, EVD, LD and the combination of EVD and LD could be surgical options for the treatment of IPIVH patients. The prognosis with these surgical procedures could be relatively favorable in most discharged patients.
